# Synthesis of Azatide Dipeptide Analogs and Their Stability and Reactivity in 98% *w*/*w* Sulfuric Acid

**DOI:** 10.3390/molecules31071196

**Published:** 2026-04-03

**Authors:** Sara Seager, Maxwell D. Seager, Ton Visser, Bartjan Koning, Jim van Wiltenburg, Martin Poelert, Janusz J. Petkowski

**Affiliations:** 1 Department of Earth, Atmospheric and Planetary Sciences, Massachusetts Institute of Technology, Cambridge, MA 02139, USA; 2 Department of Physics, Massachusetts Institute of Technology, Cambridge, MA 02139, USA; 3 Department of Aeronautics and Astronautics, Massachusetts Institute of Technology, Cambridge, MA 02139, USA; 4 Nanoplanet Consulting, Concord, MA 01742, USA; 5 Department of Chemistry and Biochemistry, Worcester Polytechnic Institute, Worcester, MA 01609, USA; 6 Symeres Netherlands BV, Kadijk 3, 9747 AT Groningen, The Netherlands; 7 Faculty of Environmental Engineering, Wroclaw University of Science and Technology, 50-370 Wroclaw, Poland; 8 JJ Scientific, 02-792 Warsaw, Poland

**Keywords:** azatides, poly-(α-aza-amino acids), peptidomimetics, concentrated sulfuric acid, ^13^C NMR, ^1^H NMR, ELSD-LCMS

## Abstract

Life as we know it depends on peptide and nucleic acid polymers built from a limited set of backbone residues, yet planetary environments beyond Earth motivate consideration of alternative chemical frameworks for genetic- and protein-like polymers. In this context, we synthesize four azatide dipeptide analogs (Ala^a^-Gly^a^ (**1**), Gly^a^-Ala^a^ (**2**), Gly^a^-Gly^a^ (**3**), and Ala^a^-Ala^a^ (**4**)) as candidate backbone motifs for non-standard biologically relevant polymers. We then systematically assess their stability and reactivity in 98% *w*/*w* sulfuric acid, a solvent relevant to Venusian cloud chemistry. We assess the stability of the azatides via ^1^H and ^13^C NMR spectroscopy supported with ELSD-LCMS. We monitor the stability of the compounds over periods from hours to two weeks at room temperature and at elevated temperatures (50–80 °C). All four azatides readily dissolve in 98% *w*/*w* D_2_SO_4_ and are generally stable at room temperature. Gly^a^-Ala^a^ (**2**) shows no detectable degradation over a two-week incubation in 98% *w*/*w* sulfuric acid. The other three azatide analogs display only minor decomposition. ELSD-LCMS measurements qualitatively confirm the NMR results, revealing only minor-to-moderate loss of parent compounds after two weeks at room temperature. At higher temperatures, representative of the lower Venusian cloud deck, the stability of the azatides decreases dramatically. All four compounds undergo significant decomposition at 50 °C and completely degrade within one to two weeks at 80 °C. Our findings indicate that azatides, despite being generally stable in concentrated sulfuric acid at room temperature, lack the thermal stability that might be required to serve as viable backbone motifs for biological polymers in environments spanning the full temperature range of Venusian clouds.

## 1. Introduction

Organic chemistry in highly concentrated sulfuric acid remains comparatively underexplored, yet it has recently emerged as an unexpectedly fertile domain. A growing body of work, extending and reinterpreting scattered studies from several decades ago (e.g., [1,2,3,4,5,6,7,8,9,10,11,12,13,14,15,16,17,18,19,20,21]), has demonstrated that a range of complex organic molecules can persist in this strongly acidic medium.

Much of the renewed attention to organic chemistry in concentrated sulfuric acid is driven by astrobiological considerations, particularly hypotheses concerning Venus’s atmosphere. Rather than the planet’s extreme surface conditions, interest has focused on the sulfuric acid cloud layers at altitudes of approximately 48–60 km, where temperatures approach those found at Earth’s surface (e.g., [22,23,24,25,26,27,28,29,30,31,32]). While the existence of complex organic chemistry does not in itself imply biology, such chemistry constitutes a necessary foundation for any potential life-like processes [33].

Recent work by Spacek and Benner has shown that a wide variety of organic reactions can arise spontaneously in concentrated sulfuric acid from simple precursors such as formaldehyde and carbon monoxide [34,35,36]. Complementing these studies, we have demonstrated the stability of multiple classes of organic building blocks in this solvent, including nucleic acid bases [37,38], amino acids [39], and dipeptides [40,41]. In addition, we have shown that simple lipid molecules not only remain intact but can self-assemble into vesicles, micelles, and other membranous structures in concentrated sulfuric acid at room temperature [42].

This recent work shows promise, yet life requires far more sophisticated molecular structures to perform biological functions. At the center of every cellular process are complex polymers, a universal requirement for any life, no matter its biochemical makeup. This requirement includes the need for genetic polymers with functional and structural properties similar to RNA and DNA [33,43], as well as the need for an “executive polymer”, an analog of proteins, that is responsible for fulfilling most of the physiological functions of the cell. Therefore, identifying genetic- and protein-like polymers that resist degradation in concentrated sulfuric acid becomes a critical step to study the possibility of life in environments where sulfuric acid, instead of water, is a dominant liquid.

We have previously demonstrated that Peptide Nucleic Acid (PNA) could be a promising candidate for a genetic polymer that is stable in concentrated sulfuric acid [44]. PNA, however, while stable at room temperature in 98% *w*/*w* sulfuric acid, decomposes above 50 °C. PNA solvolysis at high temperatures proceeds by cleavage of a single tertiary amide bond in an acetyl group linker that connects the nucleic acid base to the backbone of the polymer. The solvolysis yields two distinct products that appear to be stable to further degradation in 98% *w*/*w* sulfuric acid [44]. The thermal instability of PNA’s acetyl group linker prompts us to seek other structural analogs to DNA in parallel, in an effort to identify at least several examples of chemical motifs that could serve as stable backbone and linker residues of a genetic-like polymer in concentrated sulfuric acid [45].

One such potential backbone candidate that could serve as a structural alternative to PNA’s *N*-(2-aminoethyl) glycine (AEG) backbone, or DNA’s phosphodiester backbone, is a poly-(α-aza-amino acid), also called an azatide. In PNA, the AEG backbone closely resembles the Gly-Gly dipeptide; therefore, the azatide Gly^a^-Gly^a^-analog (**3**) and other related azatides could potentially serve as viable structural alternatives.

**Figure 1:**
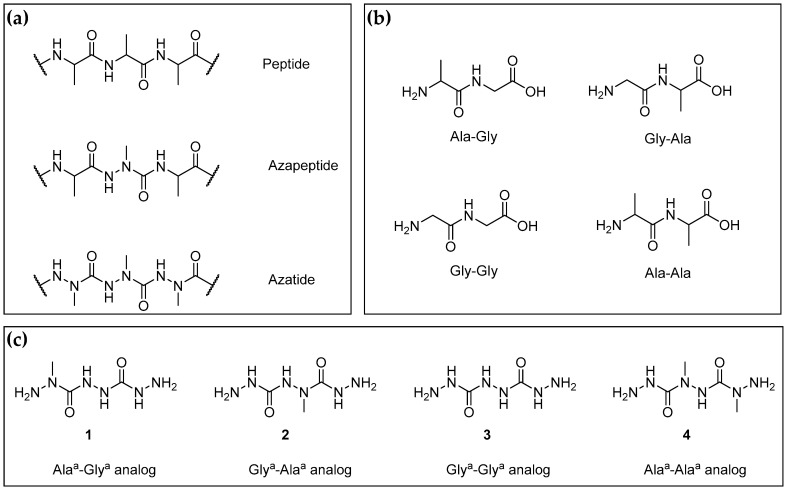
Structures of selected scaffolds of N-N bond-containing peptidomimetics. (**a**) Structures of example azapeptide and azatide peptide analogs. (**b**) Structures of the four dipeptides whose stability and solvolysis in 98% *w*/*w* sulfuric acid have been investigated before [41]. (**c**) Structures of azatide dipeptide analogs studied in this work.

Azatides, moreover, also serve as peptidomimetics [46,47], compounds that are structurally similar to polypeptide chains (Figure 1) and can therefore potentially also serve as analogs of proteins in environments where concentrated sulfuric acid is a dominant liquid. Many diverse classes of peptidomimetics (e.g., depsipeptides, ketomethylenes, thioesters, thioamides, sulfonamides, phosphoramidates, N-methyl peptides; reviewed in [48]) have become an immensely important branch of medicinal chemistry and biotechnology [49,50], predominantly acting as potent selective inhibitors of proteases. For example, azapeptide peptidomimetics have been used as inhibitors of cysteine, serine and aspartate proteases [51,52,53,54,55,56,57]. In contrast, the potential pharmacological applications of azatides have not generally been explored [58].

From the structural point of view, azatides are peptidomimetics that contain a nitrogen atom substitution at the α-carbon atom of the peptide [46,47] (Figure 1). Their characteristic feature is that, in contrast to, e.g., azapeptides, all of their α-carbons have been replaced by nitrogen. Azatides, similarly to other peptidomimetics, are generally completely resistant to protease degradation and possess unique stereochemical properties, such as the orientation of the R-group at the α-nitrogen atom, which is intermediate between the D- and L-enantiomers of the naturally occurring amino acids (Figure 1).

We have previously shown that glycylglycine (Gly-Gly), a dipeptide composed of two glycine amino acid residues, and alanylglycine (Ala-Gly), a dipeptide composed of alanine and glycine amino acids, are stable in 98% *w*/*w* concentrated sulfuric acid for many months [40,41]. We have also found that fluorination of the β-carbon in the amino acid side-chain successfully stabilizes the reactive dipeptides in 98% *w*/*w* sulfuric acid and prevents their solvolysis [41]. However, experiments on the stabilization of polypeptides to solvolysis in concentrated sulfuric acid with such a fluorination approach are synthetically challenging and expensive. It requires the substitution of every β-hydrogen with a fluorine atom in every reactive side chain of every amino acid in the polypeptide chain. We therefore simultaneously explore other molecules and chemical motifs that could serve as stable analogs of proteins in sulfuric acid biochemistry.

To evaluate azatides’ potential as key building blocks of biological polymers that are stable in concentrated sulfuric acid, we synthesize four azatide dipeptide analogs and test their stability in 98% *w*/*w* sulfuric acid. We build upon our recent successful experiments on the stabilization of Gly-Gly, Gly-Ala, Ala-Gly, and Ala-Ala dipeptides to solvolysis in 98% *w*/*w* sulfuric acid [41]. We chose their azatide dipeptide structural analogs (**1**–**4**) as the simplest homodimer and nonhomodimer examples of azatides (Figure 1). The structures of compounds **1**–**4** are simple enough that their reactivity can be followed by NMR, whereas interpreting data from more complex dipeptide analogs or longer azatides would be much more challenging, both from the synthetic as well as spectral analysis point of view.

We employ ^13^C and ^1^H Nuclear Magnetic Resonance Spectroscopy (NMR) as well as Liquid Chromatography–Mass Spectrometry (LCMS) coupled with Evaporative Light Scattering Detector (ELSD) to assess the integrity of these molecules in 98% *w*/*w* sulfuric acid at room (rt) and elevated (50–80 °C) temperatures. We monitor azatide stability over periods ranging from hours to weeks. We also describe detailed synthetic routes for all four selected azatides, both successful and unsuccessful approaches.

## 2. Results

We find that azatides **1**–**4** readily decompose at temperatures of 50–80 °C but are generally stable at room temperature (rt), with no or minor degradation after a two-week incubation in 98% *w*/*w* sulfuric acid (Table 1). We first summarize the synthesis of all four azatides (Section 2.1.). We describe the tests of the stability and reactivity of the four azatides (**1**, **2**, **3**, **4**) by NMR in Section 2.2. Finally, we follow up the NMR stability experiments with qualitative ELSD-LCMS stability tests to further support the conclusions of the NMR work (Section 2.3).

Before describing the results, we provide an overview of the reaction conditions and their relevance to Venus’ cloud chemistry and environment.

We test the stability of four azatides dissolved in D_2_SO_4_ (98 wt%, 2 wt% D_2_O). The ^1^H and ^13^C NMR spectra are measured after 1–2 h, 24 h, 7 days, and 14 days. The stability and reactivity of azatides have not been previously tested in concentrated sulfuric acid. Here, by stable, we mean that there is no detectable reactivity or degradation of the tested compounds after incubation in 98% *w*/*w* sulfuric acid for up to 14 days. We follow a similar approach and measurement time-stamps (1 h, 24 h, 7 days and 14 days) in accompanying qualitative ELSD-LCMS azatide stability experiments.

We choose 98% *w*/*w* concentrated sulfuric acid for stability experiments, following the rationale outlined in our previous related work [45,59]. In brief, there are two reasons for this choice. First, the concentration of sulfuric acid in the clouds of Venus varies with altitude, from 81% *w*/*w* acid in the top clouds, at 56.5–70 km above the surface, to 98% *w*/*w* acid in the lower cloud region (47.5–50.5 km) [60,61,62,63]. In 98% *w*/*w* the amount of water is very low, and sulfuric acid dominates the chemistry of the solvent, potentially entirely changing the expected chemical behavior of the investigated compounds as compared to more diluted acid [40,41]. Second, early experiments on the stability and reactivity of a variety of organic molecules, including proteins and peptides, often focused on similar, high concentrations of acid [10,12,16,17,18,19,20], enabling direct comparison with experiments from decades past.

We now turn to a detailed description of the results of the azatide synthesis and stability assays in 98% *w*/*w* sulfuric acid.

### 2.1. Overview of the Synthesis of Azatides ***1***, ***2***, ***3***, ***4***

**Figure 2:**
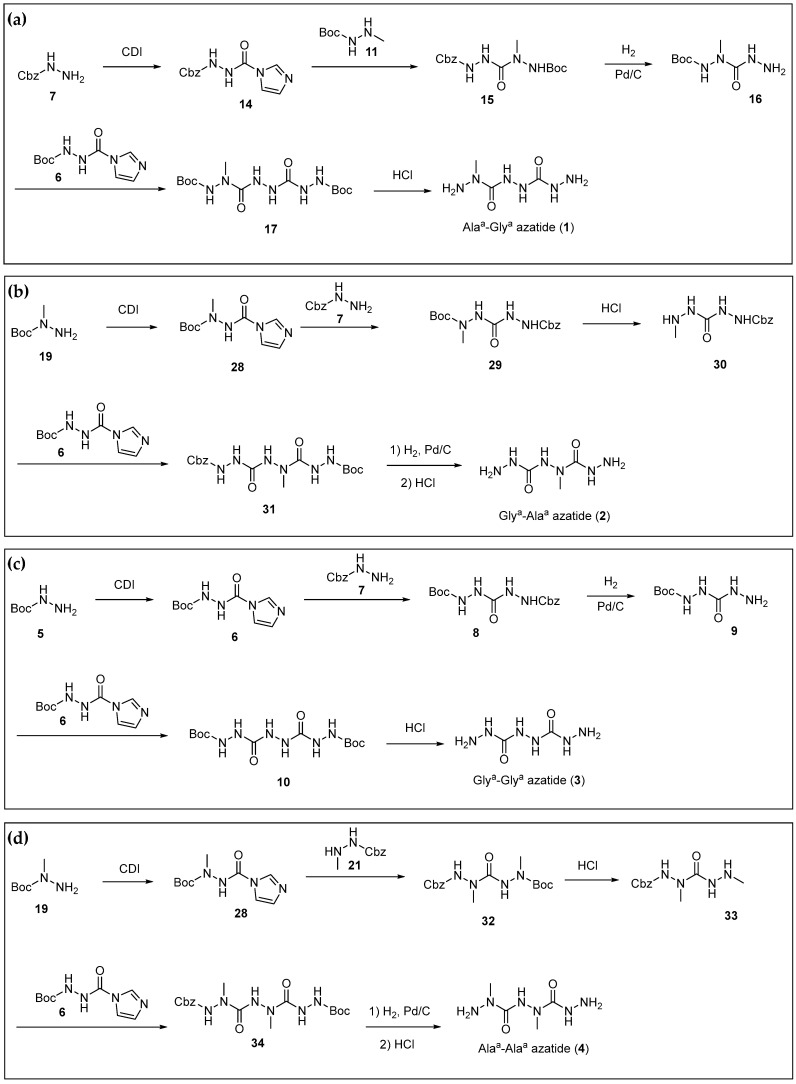
Summary of the synthesis of azatides **1**–**4**; (**a**) Schematic of the synthesis of the Ala^a^-Gly^a^ (**1**) azatide; (**b**) Schematic of the synthesis of the Gly^a^-Ala^a^ (**2**) azatide; (**c**) Schematic of the synthesis of the Gly^a^-Gly^a^ (**3**) azatide; (**d**) Schematic of the synthesis of the Ala^a^-Ala^a^ (**4**) azatide.

We successfully synthesized four azatide dipeptide analogs (**1**–**4**) using modified liquid-phase procedures with Boc and Cbz protecting groups, building upon established methods with CDI activation for hydrazine couplings [47]. Each azatide dipeptide analog required tailored synthetic strategies to overcome reactivity challenges, such as unreactive intermediates or side cyclizations. We confirmed the structural integrity of the synthesized compounds by LCMS and NMR. Below, we summarize the synthesis procedures of the four azatides (Figure 2). For the detailed description of the experimental procedures, please see Section 4, Materials and Methods.

For Ala^a^-Gly^a^ (**1**), we activated Cbz-hydrazine (**7**) with CDI to form intermediate **14** and coupled it with 1-Boc-2-methylhydrazine (**11**) to yield compound **15**. We removed the Cbz group via hydrogenation to obtain compound **16**, coupled with activated Boc-hydrazine (**6**) to form compound **17**, and performed final Boc deprotection with HCl in MeOH/IPA to afford azatide **1** in an overall moderate yield. An alternative route starting from Boc-hydrazine-carbonyl-hydrazine (**9**), activated with CDI, followed by coupling with methylhydrazine tert-butyl ester and deprotection, also successfully yielded compound **1** (see Section 4, Materials and Methods).

For Gly^a^-Ala^a^ (**2**), we activated Boc-methylhydrazine (**19**) with CDI to yield compound **28**, coupled it with Cbz-hydrazine (**7**) to give compound **29**, deprotected Boc with HCl, followed by basification to yield compound **30**. We coupled compound **30** with activated Boc-hydrazine (**6**) to form compound **31** and purified it to remove the cyclic dimer side-product contaminant. Cbz deprotection via hydrogenation and Boc deprotection with HCl resulted in compound **2** in a low to moderate yield.

For Gly^a^-Gly^a^ (**3**), we activated Boc-hydrazine (**5**) with CDI to form compound **6**, and coupled it with Cbz-hydrazine (**7**) to yield compound **8**. We removed the Cbz group via hydrogenation to obtain compound **9**. Compound **9,** coupled with compound **6** in CH_3_CN solvent, led to the precipitation of the pure compound **10**. Final Boc deprotection with HCl in MeOH/IPA afforded compound **3** in a good yield.

For Ala^a^-Ala^a^ (**4**), we activated Boc-methylhydrazine (**19**) with CDI to form compound **28**, coupled it with Cbz-2-methylhydrazine (**21**) to give compound **32**, deprotected Boc with HCl, followed by basification to yield compound **33**. Reaction of compound **33** with activated Boc-hydrazine (**6**) afforded compound **34** (purified to remove cyclic dimers). We sequentially deprotected Cbz via hydrogenation and Boc with HCl to afford compound **4** in moderate yield.

### 2.2. NMR Stability and Reactivity of Compounds ***1***, ***2***, ***3***, ***4*** in 98% w/w Sulfuric Acid

**Figure 3:**
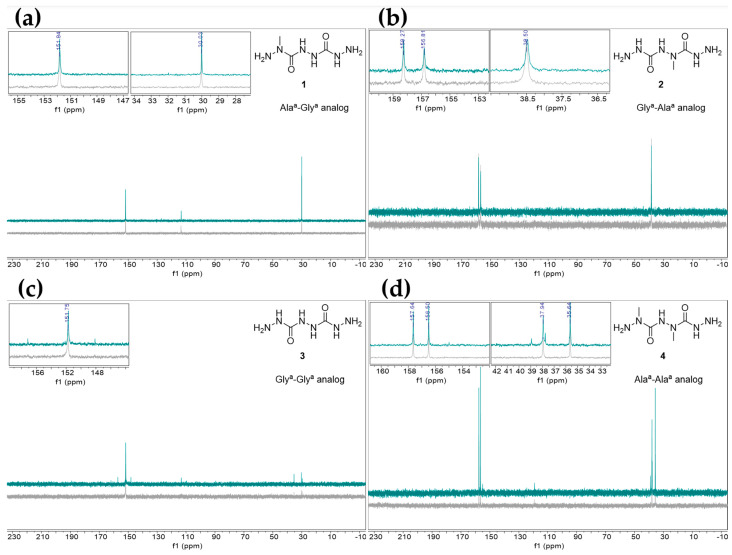
The comparison of ^13^C NMR spectra of azatides **1**–**4** in 98% D_2_SO_4_ and 2% D_2_O, by weight, at room temperature, collected after 24 h incubation (gray spectra) to spectra collected after 2-week incubation in acid; (**a**) ^13^C NMR spectra of compound **1**; (**b**) ^13^C NMR spectra of compound **2**; (**c**) ^13^C NMR spectra of compound **3**; (**d**) ^13^C NMR spectra of compound **4**. At room temperature, all four azatides are relatively stable. We see only minor degradation (few new peaks emerge) after incubation in 98% *w*/*w* sulfuric acid for two weeks.

**Figure 4:**
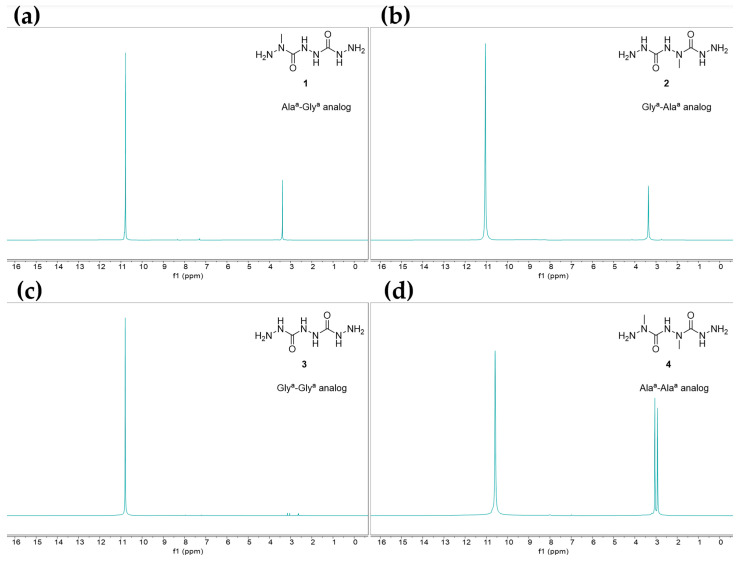
^1^H NMR spectra of azatides **1**–**4** in 98% D_2_SO_4_ and 2% D_2_O, by weight, at room temperature, collected after 2-week incubation in acid; (**a**) ^1^H NMR spectra of compound **1**; (**b**) ^1^H NMR spectra of compound **2**; (**c**) ^1^H NMR spectra of compound **3**. As expected, Gly^a^-Gly^a^ azatide does not show any proton signals. We only see trace contaminant peaks and solvent peak at around 11 ppm; (**d**) ^1^H NMR spectra of compound **4**. At room temperature, all four azatides are relatively stable. We see only minor degradation after incubation in 98% *w*/*w* sulfuric acid for two weeks.

**Figure 5:**
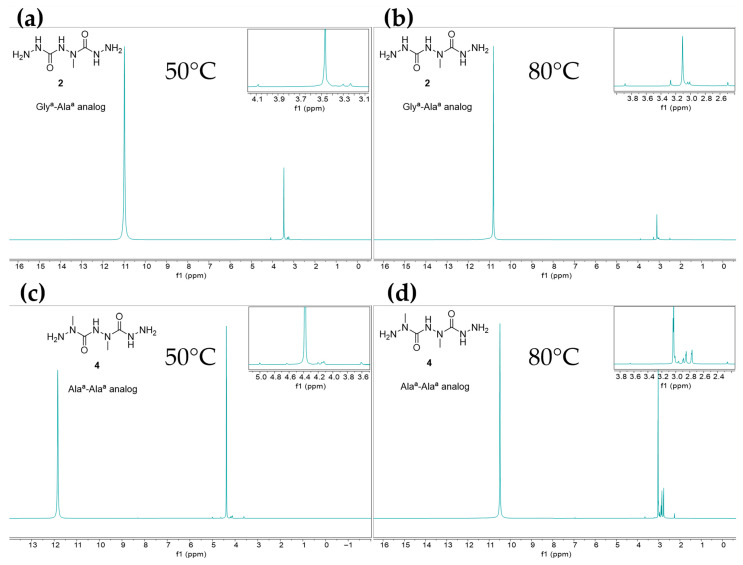
^1^H NMR spectra of azatides **2** and **4** in 98% D_2_SO_4_ and 2% D_2_O, by weight, at elevated temperatures, collected after 1-week incubation in acid; (**a**) ^1^H NMR spectra of Gly^a^-Ala^a^ azatide (**2**) at 50 °C; (**b**) ^1^H NMR spectra of Gly^a^-Ala^a^ azatide (**2**) at 80 °C; (**c**) ^1^H NMR spectra of Ala^a^-Ala^a^ azatide (**4**) at 50 °C; (**d**) ^1^H NMR spectra of Ala^a^-Ala^a^ azatide (**4**) at 80 °C. We note that samples were difficult to shim. Therefore, for Ala^a^-Ala^a^ azatide, we selected D-TFA for shimming and locking. We see new peaks emerge after incubation of azatides in acid at high temperature. These new peaks indicate that azatides are not stable and degrade at high temperatures in concentrated sulfuric acid.

All four tested azatides are readily soluble in 98% *w*/*w* sulfuric acid. Compound **2** appears to be completely stable for at least two weeks in 98% *w*/*w* D_2_SO_4_ at rt, with no signs of detectable degradation. Azatides **1**, **3**, **4** exhibit only minor degradation at rt after two weeks of incubation in acid (Table 1, Figure 3 and Figure 4), as shown by the ^1^H and ^13^C NMR spectra of compounds (**1**–**4**) not significantly changing over the span of the two-week incubation in 98% *w*/*w* sulfuric acid at rt. All four azatides, however, readily decompose at high temperatures (50–80 °C) (Table 1, Figure 5).

We begin by describing the detailed NMR assignment of the four azatides, focusing on the spectra collected after a 2-week incubation in 98% *w*/*w* sulfuric acid at rt.

For compound **1**, in the ^13^C NMR spectrum (Figure 3a), we find a single peak that corresponds to the two carbonyl carbons in the Ala^a^-Gly^a^ azatide structure (peak δ 151.84). We note that the single peak that corresponds to the two carbonyl carbons has a visible shoulder, which is consistent with a near-perfect overlap and merger of the two carbonyl peak NMR signals. In the D_2_O solvent, these two carbonyl peaks are well separated (peaks δ 159.17, 158.50). We also see a clear peak corresponding to the methyl carbon in the Ala^a^-analog residue (peak δ 30.03, with a matching peak δ 36.65 in D_2_O). Finally, the signal at around 113 ppm seems to be the artifactual “ghost peak”, usually called “Central Spike”, or “DC Offset peak”, which can sometimes be visible in the middle of the spectrum [64], and is not a sign of reactivity of compound **1**.

Over time, the Ala^a^-Gly^a^ azatide (**1**) shows only very minor degradation. After a two-week incubation in 98% *w*/*w* D_2_SO_4,_ we see only one new emergent trace peak at 32 ppm. The ^1^H NMR spectra are also consistent with compound **1** being generally stable at rt for at least 2 weeks (Figure 4a).

**Figure 6:**
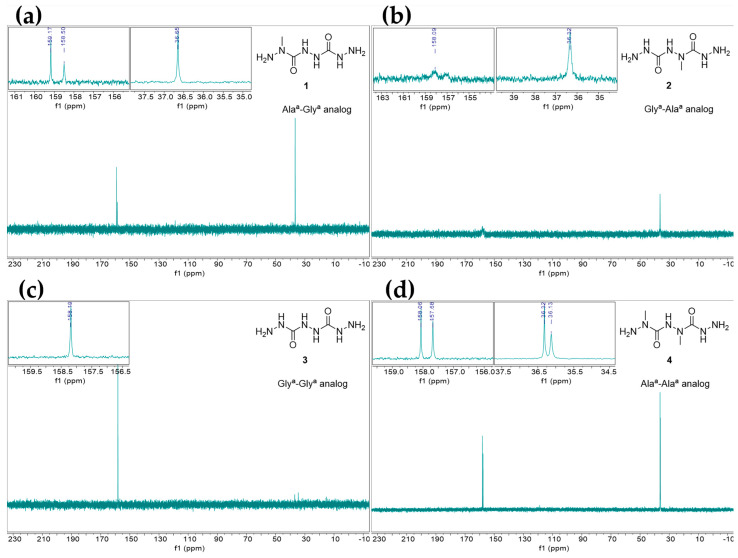
^13^C NMR spectra of azatides **1**–**4** in D_2_O, at room temperature; (**a**) ^13^C NMR spectra of compound **1**; (**b**) ^13^C NMR spectra of compound **2**; Note that peaks corresponding to carbonyl carbons are flattened and broadened in D_2_O. They do separate well, however, in 98% *w*/*w* D_2_SO_4_ (Figure 3b) (**c**) ^13^C NMR spectra of compound **3**. (**d**) ^13^C NMR spectra of compound **4**. At room temperature, in D_2_O, all four azatides are stable.

**Figure 7:**
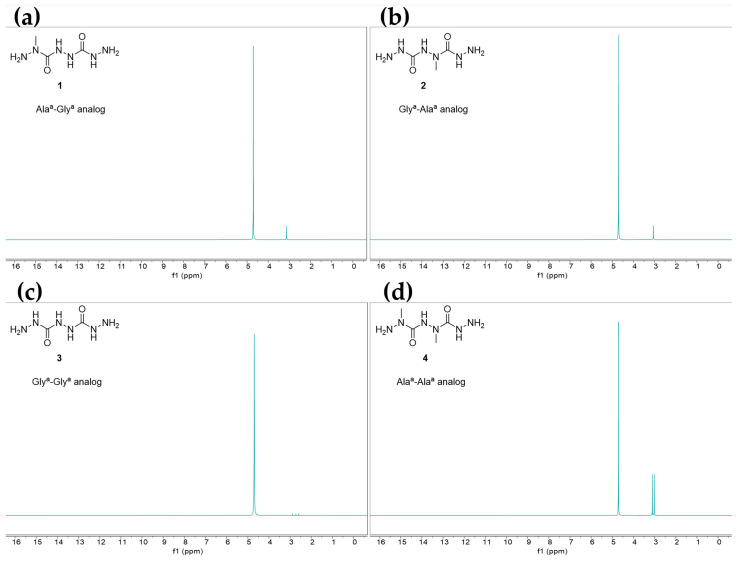
^1^H NMR spectra of azatides **1**–**4** in D_2_O, at room temperature; (**a**) ^1^H NMR spectra of compound **1**; (**b**) ^1^H NMR spectra of compound **2**; (**c**) ^1^H NMR spectra of compound **3**. As expected, Gly^a^-Gly^a^ azatide does not show any proton signals. We only trace contaminant peaks and D_2_O solvent peak at around 4.7 ppm. (**d**) ^1^H NMR spectra of compound **4**. At room temperature, in D_2_O, all four azatides are stable.

For compound **2**’s ^13^C NMR spectrum (Figure 3b), we find two peaks that correspond to the two carbonyl carbons in the azatide structure (peaks δ 158.27, 156.81). We also see a clear peak corresponding to the methyl carbon in the Ala^a^-analog residue (peak δ 38.50). The ^1^H NMR spectra are also consistent with compound **2** being stable at rt for at least 2 weeks (Figure 4b). We observe no degradation of the azatide Gly^a^-Ala^a^ (**2**) at rt. No new peaks emerge over the span of the 2-week incubation, confirming the overall stability of compound **2** in 98% *w*/*w* sulfuric acid. We also note that the chemical shift trends for ^1^H and ^13^C NMR spectra collected in 98% *w*/*w* D_2_SO_4_ agree with values of ^13^C NMR (peaks δ 158.09, 157.08, 36.32) and ^1^H NMR collected in D_2_O solvent (Figure 6 and Figure 7).

The Gly^a^-Gly^a^ azatide analog (**3**) appears to be the most reactive of the four tested compounds at rt. In the ^13^C NMR spectrum (Figure 3c), we find a single peak that corresponds to the two symmetric carbonyl carbons in the Gly^a^-Gly^a^ azatide structure (peak δ 151.75). We also see a series of peaks around 30 ppm in the ^13^C NMR spectrum that correspond to impurities. The impurities visible in the ^13^C NMR of the Gly^a^-Gly^a^ azatide could arise from traces of IPA and MeOH solvents used in the synthesis. These solvents could also readily react in 98% *w*/*w* D_2_SO_4_ and lead to the formation of additional, more complex side-products and impurities. As expected, the ^1^H NMR spectra do not show any proton signals apart from the aforementioned impurities and the dominant solvent peak (compound **3** does not have any C-H protons in its structure) (Figure 4c). We observe minor degradation of the azatide Gly^a^-Gly^a^ (**3**) at rt after one week of incubation in 98% *w*/*w* D_2_SO_4_. We also note that the chemical shift trends for ^13^C NMR spectra collected in 98% *w*/*w* D_2_SO_4_ agree with values of ^13^C NMR in D_2_O solvent (peak δ 158.19) (Figure 6 and Figure 7).

For compound **4**, in the ^13^C NMR spectrum (Figure 3d), we find two peaks that correspond to the two carbonyl carbons in the azatide structure (peaks δ 157.64, 156.50). We also see two clear peaks corresponding to the two methyl carbons in the Ala^a^-Ala^a^ azatide (**4**) (peaks δ 37.94, 35.64). We do observe minor degradation of the azatide Ala^a^-Ala^a^ (**4**) at rt. At least two new peaks emerge over the span of the 2-week incubation in 98% *w*/*w* sulfuric acid. In the high field region, one of the methyl groups seems to degrade, and a new signal emerges at around 39 ppm. At low field, a new signal at 155 ppm shows up. The ^1^H NMR spectra are also consistent with compound **4** being relatively stable at rt (Figure 4d). We also note that the chemical shift trends for ^1^H and ^13^C NMR spectra collected in 98% *w*/*w* D_2_SO_4_ agree with values of ^13^C NMR (peaks δ 158.06, 157.68, 36.32, 36.13) and ^1^H NMR collected in the D_2_O solvent (Figure 6 and Figure 7).

As an aside, we note that azatides **1**–**4** have been prepared as HCl salts (see Section 4). During dissolution of the tested compounds in concentrated sulfuric acid, HCl gas is released. The release of the HCl gas does not affect the stability of the tested compounds.

We now turn to an assessment of the stability of azatides **1**–**4** in 98% *w*/*w* D_2_SO_4_ at elevated temperatures. The thermal stability test is important, as the temperature range in the acidic Venusian cloud deck spans approx. 100 °C. In the top cloud levels (~60 km altitude above the surface), the temperatures fall below 0 °C, while at the bottom of the clouds, at 48 km altitude, the temperatures reach 80–90 °C [65]. None of the tested compounds survives such high temperatures after incubation in 98% *w*/*w* sulfuric acid. For example, at 50 °C compounds **2** and **4** show moderate to large degradation, while at 80 °C we see their rapid and significant decomposition that eventually leads to their complete destruction. The Ala^a^-Ala^a^ azatide (**4**) degrades much more readily at high temperatures than the Gly^a^-Ala^a^ azatide (**2**) (Figure 5). This finding is substantiated by the results from the LCMS-ELSD stability measurements. We discuss the LCMS-ELSD results next.

### 2.3. LCMS-ELSD Stability Tests of Compounds ***1***, ***2***, ***3***, ***4*** in 98% w/w Sulfuric Acid

**Figure 8:**
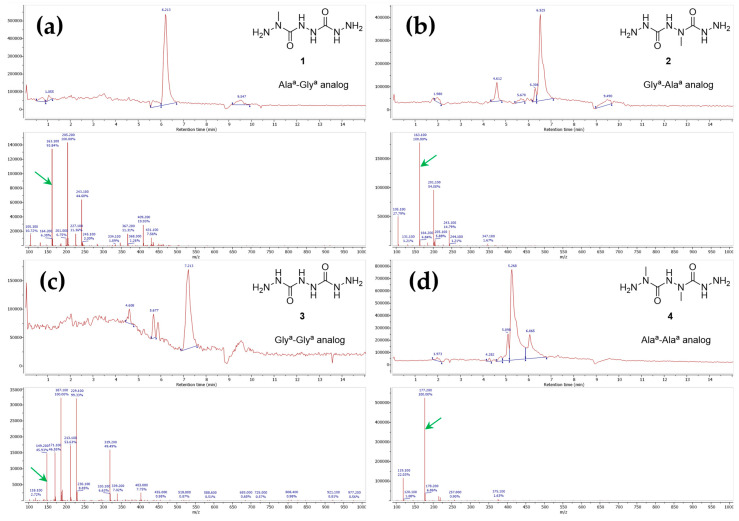
LCMS analysis of four azatide compounds (**1**–**4**) incubated for two weeks at room temperature in 98% *w*/*w* sulfuric acid. The individual panels show representative total ion chromatogram (TIC) and positive electrospray ionization (ESI) mass spectrum obtained by reversed-phase LCMS of compounds **1**–**4**. The TIC (upper panels) displays retention time (min) on the *x*-axis and total ion intensity on the *y*-axis. The corresponding ESI-MS spectra (lower panels) show *m*/*z* (mass to charge ratio) on the *x*-axis and relative abundance (%), where the intensity of each ion is normalized to the base (tallest) peak at 100%, on the *y*-axis. Green arrows point to the *m*/*z* peak corresponding to the target azatide compound. Additional peaks are unidentified; they are most likely, e.g., method-specific artifacts from solvent adducts. (**a**) LCMS analysis of azatide compound **1**. (**b**) LCMS analysis of azatide compound **2**. (**c**) LCMS analysis of azatide compound **3**. (**d**) LCMS analysis of azatide compound **4**. Overall, the LCMS results demonstrate high purity and stability of the tested azatides at rt. Out of the four compounds, **3** is the least pure and exhibits minor to moderate degradation in concentrated sulfuric acid. Compounds **1**, **2**, **4** show no significant degradation at rt after two-week incubation in sulfuric acid.

We use the LCMS-ELSD as a supportive technique to monitor the stability of the azatides in 98% *w*/*w* sulfuric acid. The qualitative ELSD stability assessment for all four azatides largely agrees with the NMR results presented in Section 2.2. LCMS-ELSD analysis shows that all four tested azatides are generally stable in 98% *w*/*w* sulfuric acid at rt, with only minor degradation visible after a two-week incubation (Figure 8).

At room temperature, Ala^a^-Gly^a^ azatide (**1**) is generally stable. We estimated the stability of the compound from the changes in the ELSD signal. For example, after 2 weeks at rt the LCMS peak area corresponding to the target compound **1** drops to 86%, down from the starting value of 98.8%, indicating minor-to-moderate degradation. Such a result is consistent with the NMR studies. At elevated temperatures, 50 °C and 80 °C, the compound starts to degrade more rapidly. Ala^a^-Gly^a^ azatide (**1**) fully decomposes at 80 °C after a two-week incubation in 98% *w*/*w* D_2_SO_4_ (Table 2).

At room temperature, Gly^a^-Ala^a^ azatide (**2**) is stable. The stability of compound **2** agrees with the NMR studies. At 50 °C, the Gly^a^-Ala^a^ azatide (**2**) is relatively stable for 2 weeks (Table 3). At 80 °C, the compound starts to degrade rapidly, and Ala^a^-Gly^a^ azatide (**2**) fully decomposes at 80 °C after one week of incubation in 98% *w*/*w* D_2_SO_4_.

At room temperature, Gly^a^-Gly^a^ azatide (**3**) is generally stable for 2 weeks. We saw only a minor degradation after one week of incubation in 98% *w*/*w* sulfuric acid. Similarly to the NMR results, we detected impurities in the tested sample. At 50 °C, the compound starts to degrade, and at 80 °C, the azatide seems to have decomposed completely (Table 4).

At room temperature, Ala^a^-Ala^a^ azatide (**4**) is stable. The stability of compound **4** largely agrees with the NMR studies. At elevated temperatures, 50 °C and 80 °C, the compound starts to degrade rapidly. Ala^a^-Ala^a^ azatide (**4**) fully decomposes after one week in 98% *w*/*w* D_2_SO_4_ at 80 °C. At 50 °C, Ala^a^-Ala^a^ azatide (**4**) also degrades rapidly, and we observed significant degradation after two weeks in 98% *w*/*w* D_2_SO_4_ (Table 5).

We conclude this section with a discussion of the caveats and limitations of the LCMS-ELSD stability assay. Our LCMS-ELSD stability assessment is qualitative due to the inherent limitations of the method. One significant limitation is that the azatides do not absorb UV light and, as such, are difficult to analyze chromatographically. The Evaporative Light Scattering Detection (ELSD) detector is one of the few detectors that can be coupled with high-performance liquid chromatography (HPLC) for the detection and analysis of compounds that do not absorb UV light. ELSD works by nebulizing the liquid sample of the target compound into a fine aerosol, evaporating the solvent, and measuring the light scattered by the remaining non-volatile particles. The detected scattered light is converted into a signal that is proportional to the amount of the studied compound. In the case of the tested azatides, however, ELSD does not give a very precise measurement of the purity of the tested compound, making our ELSD stability assessment largely qualitative. The qualitative nature of our stability analysis has several causes.

First, we performed the stability assay in concentrated sulfuric acid. The presence of acid requires the samples to be quenched with a buffer (3M NH_4_OAc) for the analysis. We chose 3M NH_4_OAc as an acid-quenching buffer because it does not affect the reactivity and stability of the tested compounds. The buffer, however, interferes with ELSD and prevents precise quantitative measurements.

Secondly, we could only successfully synthesize azatides **1**–**4** as HCl salts (see Section 4, Materials and Methods). The HCl itself gives a retention time peak at 5.7 min, interfering with the ELSD method. We estimated this interference by measuring a blank sample with HCl (see Supplementary Data Set S1).

Thirdly, we recorded a significant shift in the peak retention time for compounds **2** and **4** when the starting materials were mixed with sulfuric acid. Moreover, the tested azatides had broad peaks after dissolving in concentrated sulfuric acid and quenching with the buffer. Such broadening of peaks also contributes to the largely qualitative nature of the azatides’ ELSD stability estimates.

Finally, the compounds are very hygroscopic. The highly hygroscopic nature of the tested azatides (especially **2** and **4**) means that preparing the material for the ELSD stability test cannot effectively be done without attracting water from the surrounding environment. This additional water might have influenced the outcome of the stability tests.

## 3. Discussion

Our work is part of a greater effort toward the design and synthesis of alternative structural frameworks for biological polymers capable of withstanding a concentrated sulfuric acid environment, including a counterpart to DNA as a genetic polymer and counterparts to protein polypeptide chains as “executive polymers.” DNA itself rapidly degrades in sulfuric acid due to the solvolysis of deoxyribose residues and the phosphodiester backbone of the polymer. Proteins, in contrast, exhibit diverse chemical behavior in concentrated sulfuric acid, from rapid solvolysis to distinct amino acid side chain modifications. Such reactivity warrants the search and design of a new backbone-linker residue for a genetic polymer (substituting the reactive deoxyribose) as well as potential candidates for peptide backbone analogs. Once these potential candidate chemical motifs have been identified, their stability and reactivity in concentrated sulfuric acid need to be tested.

In this work, we have explored azatides as potential candidates for an alternative backbone of genetic polymers and as a potential alternative for a peptide linker in polymers analogous to proteins. Our results show that azatides are not particularly promising candidates for these roles. While azatides are relatively stable at rt, with only minor degradation visible in 98% *w*/*w* sulfuric acid, they rapidly degrade at higher temperatures. As the temperature of the clouds of Venus ranges between approx. 80 °C at the bottom of the clouds to below 0 °C at the top of the clouds, any candidate structural analog should withstand sulfuric acid not only at rt but also at the higher temperatures encountered in the lower clouds.

We also note that azatides are not the exact structural twins of polypeptides. Azatides and peptides have significant structural differences that could potentially affect their biological functionality. The substitution of the α-carbon to nitrogen in azatides (i.e., replacing a tetrahedral center with a planar nitrogen) leads to the loss of an easily rotatable Cα−C bond and overall changes in the structure and dynamics of the polymer backbone. Azatides are therefore expected to be more rigid and to have significantly higher rotational barriers than conventional peptides [66]. The nitrogen-rich backbone of the azatide chain introduces a series of repulsive electrostatic interactions between lone pairs of nitrogen atoms. Such repulsive interactions do not exist in the N−Cα bond of conventional peptides. These electrostatic barriers have to be overcome to rotate the N−N bond. Secondly, the change from α-carbon to nitrogen removes the chirality of the polymer. These differences likely have significant consequences for the overall structure and flexibility of azatides, as compared to their conventional peptide counterparts. Whether the expected rigidity of the azatide polymer would be an insurmountable obstacle for the folding of the polymer and the adoption of the secondary structure in solution is unknown, but could be a focus of future dedicated theoretical or modeling work.

## 4. Materials and Methods

**Scheme 1:**
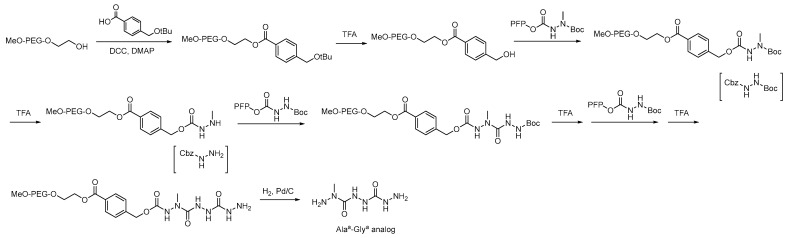
Polymer liquid phase synthesis of the Ala^a^-Gly^a^ analog (**1**).

The four azatides (**1**–**4**) that we synthesized have a C-terminal hydrazide group instead of the more common carboxylic acid cap. We decided that the carboxylic acid as a terminal group could be quite labile in such dipeptide analogs due to its ability to expel carbon dioxide (analogous to *N*-carbamate deprotection reaction). To prevent such potential reactivity in concentrated sulfuric acid, we decided to use azatides with a hydrazide C-terminus instead. Short azatide peptide analogs have been synthesized before using polymer-supported liquid phase synthesis (Scheme 1) (see Ref. [47]). This method uses a soluble linear homopolymer (MPEG), which serves as a terminal protecting group for the peptide to be synthesized. The essence of this technology is that it avoids the difficulties encountered in solid phase synthesis but preserves the positive aspects of solution phase synthesis.

Instead of working on a solid resin, as is common practice in standard peptide synthesis, this polymer ensures a fully soluble reaction mixture. Because of the unique physical properties of the MPEG polymer, each coupling/deprotection step can be purified by precipitation of the modified polymer. It also allows the reactions to be monitored by NMR. We follow and build upon the reaction sequence from Ref. [47] in the *N*-to-*C*-terminal construction of the azatides, noting that each azatide analog needed its own tailored strategy to synthesize them with a good yield and purity.

The scheme above (Scheme 1) indicates that three iterative coupling/deprotection sequences are needed to prepare each proposed analog. Each analog requires the use of a specific (methylated) hydrazine building block. Previous work [47] suggests that only the primary amine of a Boc-protected alkylhydrazine can be activated with the pentafluoro phenol carbonate reagent, which led the authors to conclude that it is likely the isocyanate that is the actual activated intermediate and not the pentafluoro phenol carbamate.

An alternative to the use of the MPEG polymer would be to use a Cbz protecting group. The deprotection conditions would be the same, but the potential advantage of precipitation of the MPEG polymer would be lost, and conventional isolation and purification, likely using RP-HLPC, would be required.

We confirmed the structural integrity of the synthesized compounds with Liquid Chromatography–Mass Spectrometry (LCMS) and NMR spectroscopy. We used MNova software, version: 14.3.0-30573 (Mestrelab Research, Barcelona, Spain) to process and analyze the NMR data [67].

The original data for all NMR, ELSD and LCMS experiments for all products, as well as for synthetic intermediates, are available for download as Supplementary Datasets from Zenodo at https://zenodo.org/records/18442323.

### 4.1. Synthesis of the Ala^a^-Gly^a^-Analog (***1***)

**Scheme 2:**
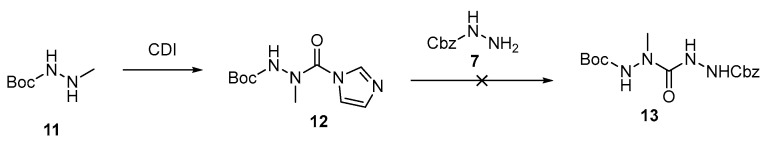
Synthesis of Ala^a^-Gly^a^-analog (**1**).

The easiest approach to preparing the Ala^a^-Gly^a^-analog would be to react 1-Boc-2-methylhydrazine **11** with CDI and then couple Cbz-hydrazine **13**. Subsequent deprotection and reaction with another hydrazine would provide the Ala^a^-Gly^a^-analog **1** (Scheme 2).

**Scheme 3:**
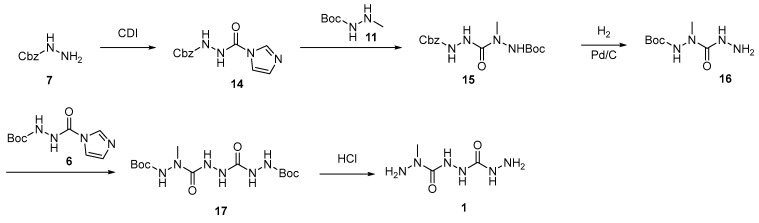
First alternative synthesis of Ala^a^-Gly^a^-analog (**1**).

However, after activating N-methylhydrzine **11** with CDI, the coupling with Cbz-hydrazine did not proceed. This challenge has also been described in the literature for the pentafluoro phenol carbamate [47]. Another approach was implemented, in which the N-methylhydrazine was reacted with the activated Cbz-hydrazine **14** (Scheme 3).

Cbz-hydrazine **7** was reacted with CDI to form the active intermediate **14**. We note that compared to the Boc-hydrazine addition, this reaction also yielded a double addition of the hydrazine to CDI. 1-Boc-2-methyl-hydrazine **11** was added to the activated intermediate **14**, and compound **15** was obtained and purified. Cbz-deprotection with H_2_ and Pd/C afforded compound **16**. Reaction of compound **16** with compound **6** gave compound **17**. Compound **17** could be purified by column chromatography. Final deprotection with HCl in MeOH/IPA afforded compound **1**. The last traces of organic solvents were removed by dissolving compound **1** in a small amount of water and lyophilizing.

**Scheme 4:**
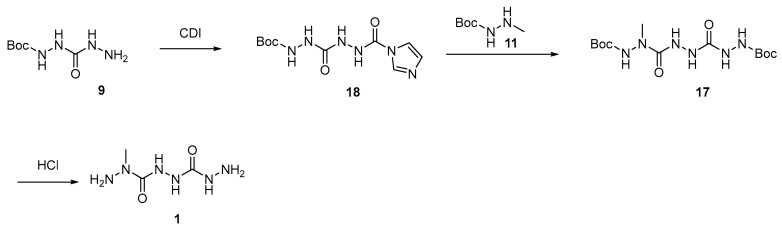
Second alternative synthesis of Ala^a^-Gly^a^-analog (**1**).

Another approach toward the Ala^a^-Gly^a^-analog (**1**) was also tested, which could be useful for the synthesis of the other azatide targets (Scheme 4).

A batch of compound **9** was prepared, and a better purification method was found: trituration in DCM gives pure product in 83% yield. This product was activated with CDI in CH_3_CN, and after full conversion, 1-Boc-2-methylhydrazine **11** was added. This afforded crude compound **17**. After purification by column chromatography, compound **17** was deprotected with HCl in IPA to afford another batch of compound **1**.

Below, we provide a detailed description of the synthetic procedures for key intermediates utilized in the synthesis of target azatide (**1**).

#### 4.1.1. Synthesis of Tert-Butyl 2-(2-((Benzyloxy)carbonyl)hydrazine-1-carbonyl)-2-methylhydrazine-1-carboxylate (**15**)

CDI (2.0 g, 1 equiv., 12 mmol) was dissolved in 10 mL of DMF, and benzyl hydrazinecarboxylate (2.0 g, 1 equiv., 12 mmol) in 5 mL of DMF was added dropwise at room temperature. The mixture was stirred for 1.5 h at room temperature. Tert-butyl 2-methylhydrazine-1-carboxylate (1.8 g, 1 equiv., 12 mmol) was added, and the mixture was stirred overnight. The mixture was diluted with 20 mL of water and extracted with MTBE (2 × 25 mL). The combined organic layers were washed with water (25 mL), dried over Na_2_SO_4_ and concentrated, affording 3.9 g of white foam. The crude material was purified by column chromatography (silica (80 g); Heptanes/EtOAc (0–80%)). Fractions 53–70 were combined and concentrated, affording tert-butyl 2-(2-((benzyloxy)carbonyl)hydrazine-1-carbonyl)-2-methylhydrazine-1-carboxylate (**15**) (2.2 g, 6.5 mmol, 53%) as a white solid.

#### 4.1.2. Synthesis of Tert-Butyl 2-(Hydrazinecarbonyl)-2-methylhydrazine-1-carboxylate (**16**)

A total of 5 mL of MeOH was added to Pd/C (0.35 g, 10% wt, 0.05 equiv., 0.33 mmol). Tert-butyl 2-(2-((benzyloxy)carbonyl)hydrazine-1-carbonyl)-2-methylhydrazine-1-carboxylate (**15**) (2.2 g, 1 equiv. 6.5 mmol) was added in 25 mL of MeOH, and the mixture was stirred under an H_2_-atmosphere for 1 h. The mixture was filtered over Celite and concentrated, affording tert-butyl 2-(hydrazinecarbonyl)-2-methylhydrazine-1-carboxylate (**16**) (1.3 g, 6.4 mmol, 98%) as a white solid.

#### 4.1.3. Synthesis of Tert-Butyl 2-(2-(2-(Tert-butoxycarbonyl)hydrazine-1-carbonyl)hydrazine-1-carbonyl)-2-methylhydrazine-1-carboxylate (**17**)

Tert-butyl 2-(1H-imidazole-1-carbonyl)hydrazine-1-carboxylate (**6**) (270 mg, 1 equiv., 1.19 mmol) was dissolved in 10 mL of CH_3_CN, and tert-butyl 2-(hydrazinecarbonyl)-2-methylhydrazine-1-carboxylate (**16**) (244 mg, 1 equiv., 1.19 mmol) was added. The mixture was stirred for 18 h at room temperature. The mixture was concentrated, affording an oil.

A total of 10 mL of MTBE was added. The material dissolved partially. Heptanes were added to crash out the material. The solvents were concentrated again, affording a white solid. The crude material was purified by column chromatography (silica (25 g); DCM/MeOH (0–8%)). Fractions 42–45 were concentrated, affording 200 mg of the product as a white foam. Fractions 46–55 were concentrated, affording 300 mg of the product as white foam.

#### 4.1.4. Synthesis of the Ala^a^-Gly^a^-Analog (**1**)

Tert-butyl 2-(2-(2-(tert-butoxycarbonyl)hydrazine-1-carbonyl)hydrazine-1-carbonyl)-2-methylhydrazine-1-carboxylate (**17**) (300 mg, 1 equiv., 828 μmol) was dissolved in 3 mL of MeOH, and 10 mL of 5 N HCl in IPA was added. The mixture was stirred for 4 h. A white precipitate formed. The white precipitate was collected by filtration, rinsed with IPA (1 mL) and dried in vacuo. The filtrate was concentrated in vacuo. The HPLCs of both fractions were similar. Both fractions were dissolved in 5 mL of water and lyophilized. The precipitate that was filtered off provided pure N’-hydrazinecarbonyl-N-methylmethanedihydrazide dihydrochloride (95 mg, 0.40 mmol, 49%) as a white solid. The concentrated filtrate contained residual imidazole.

#### 4.1.5. Synthesis of Tert-Butyl 2-(2-(2-(Tert-butoxycarbonyl)hydrazine-1-carbonyl)hydrazine-1-carbonyl)-2-methylhydrazine-1-carboxylate (**17**)—Alternative Route

CDI (0.94 g, 1.1 equiv., 5.8 mmol) was dissolved in 25 mL of CH_3_CN, and a solution of tert-butyl 2-(hydrazinecarbonyl)hydrazine-1-carboxylate (**9**) (1.0 g, 1 equiv., 5.3 mmol) in 25 mL of CH_3_CN was slowly added over 15 min. The mixture was stirred for 1 h. 2-methylhydrazine carboxylic acid tert-butylester (**11**) (0.85 g, 1.1 equiv., 5.8 mmol) was added, and the mixture was stirred for 3 h. The crude material was purified by column chromatography (silica (40 g); DCM/MeOH (0–8%)). Fractions 66–105 were concentrated, affording tert-butyl 2-(2-(2-(tert-butoxycarbonyl)hydrazine-1-carbonyl)hydrazine-1-carbonyl)-2-methylhydrazine-1-carboxylate (**17**) (900 mg, 2.48 mmol, 47%) as a white foam.

#### 4.1.6. Synthesis of the Ala^a^-Gly^a^-Analog (**1**)—Alternative Route

Tert-butyl 2-(2-(2-(tert-butoxycarbonyl)hydrazine-1-carbonyl)hydrazine-1-carbonyl)-2-methylhydrazine-1-carboxylate (**17**) (900 mg, 1 equiv., 2.48 mmol) was dissolved in 3 mL of MeOH, and 25 mL of 5 N HCl in IPA was added. The mixture was stirred for 4 h and concentrated. The residue was triturated in DCM and filtered off. The residue was triturated in 10 mL of IPA and stirred for 2 h. The white solid was collected by centrifuging. The solid was thoroughly mixed with 2 mL of IPA again and centrifuged again. Then the solid was taken up in water (3 mL) and lyophilized. The material was dissolved in 4 mL of water again and lyophilized, affording 340 mg of the product as an off-white solid free of organic solvents.

### 4.2. Synthesis of the Gly^a^-Ala^a^-Analog (***2***)

**Scheme 5:**
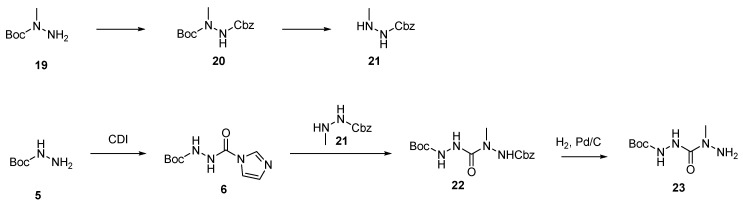
Synthesis of a common intermediate **23**.

A route to prepare the Gly^a^-Ala^a^ and the Ala^a^-Ala^a^-analogs could, in principle, run through the common intermediate **23** (Scheme 5). Cbz-deprotection and reaction with the activated hydrazine would provide the Gly^a^-Ala^a^-analog. Boc-deprotection and reaction with an activated methyl-hydrazine would provide the Ala^a^-Ala^a^-analog.

For this approach, 1-Cbz-2-methylhydrazine (**21**) needs to be prepared. 1-Boc-1-methyl hydrazine (**19**) was Cbz-protected, and subsequent Boc-deprotection afforded 1-Cbz-2-methylhydrazine **21**. Reaction of 1-Cbz-2-methylhydrazine (**21**) with activated hydrazide **6** afforded compound **22**. Cbz deprotection with H_2_ and Pd/C afforded compound **23**.

**Scheme 6:**
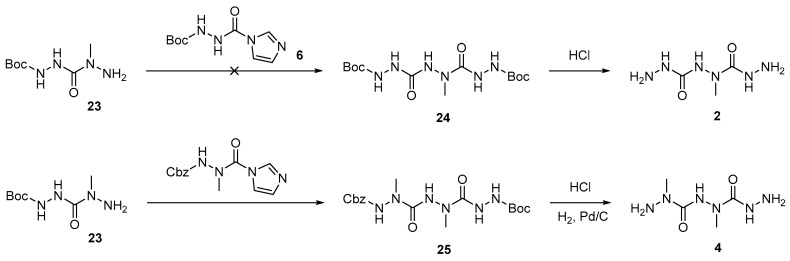
Proposed synthesis of Gly^a^-Ala^a^ (**2**) and Ala^a^-Ala^a^-analog (**4**).

The subsequent reaction of compound **23** with activated hydrazide **6** was expected to provide compound **24,** and reaction with the activated 1-Cbz-2-methyl hydrazide was expected to provide compound **25** (Scheme 6). It was anticipated that activation of the secondary amine might give an inactive intermediate.

**Scheme 7:**
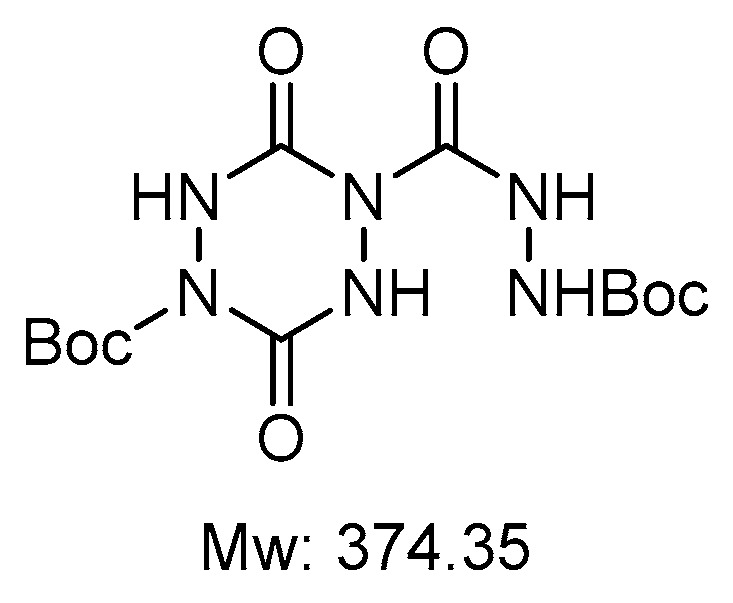
Cyclized side-product.

Freshly activated hydrazide **6** was prepared, and compound **23** was added. After stirring overnight, unfortunately, no desired product was obtained. Compound **6** seems unreactive under these conditions without the base present. Therefore, a catalytic amount of 4-DMAP was added. After stirring over the weekend, a precipitate was formed. HPLC showed only a trace of the desired product. The majority of the product seemed to be a cyclized bis-hydrazine that reacted with the activated hydrazine **6** (Scheme 7). It was found that when a base is used in the coupling reactions, the activated hydrazine, like **6,** cyclizes by an intermolecular attack on the imidazole-carbonyl. The formed bis-hydrazide then reacts with another equivalent of activated hydrazine **6**.

**Scheme 8:**
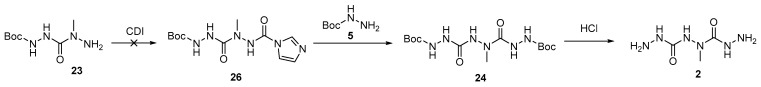
Proposed alternative synthesis of Gly^a^-Ala^a^-analog (**2**).

Since the first approach only showed traces of the desired product in HPLC, another approach was tried (Scheme 8). Compound **23** was treated with CDI and stirred for 1 h. So far, in all cases, the hydrazine has needed less than 1 h to fully react with CDI. In the case of hydrazide **23,** no reaction occurred at all. Pyridine was added, but this did not lead to conversion. In the end, Cbz-Cl was added to check if the hydrazide was reactive at all. After stirring overnight, full conversion back to compound **22** was observed. The desired compound had been prepared, but was simply not reactive enough for a reaction with CDI. The addition of a base to enhance the reaction would most likely have led to side reactions like cyclization.

**Scheme 9:**
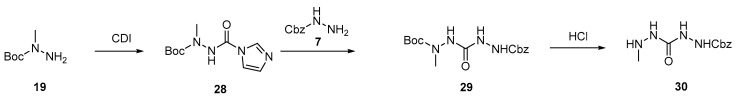
Preparation of intermediate **30**.

Therefore, a new approach was conceived, starting from 1-Boc-1-methylhydrazine (**19**), to prepare intermediate **30** (Scheme 9).

1-Boc-1-methylhydrazine (**19**) was treated with CDI, and the activated hydrazide **28** was obtained. A trace of bis-hydrazide was observed. Then Cbz-hydrazine (**7**) was added, and the desired product, **29**, was obtained after column chromatography. Boc deprotection with HCl gave a clean conversion, and the product was isolated by concentration of the solvent as an HCl salt. In the next step, we attempted to produce free amine **30**, and treatment with a minimal amount of 1 N NaOH afforded this free amine.

**Scheme 10:**
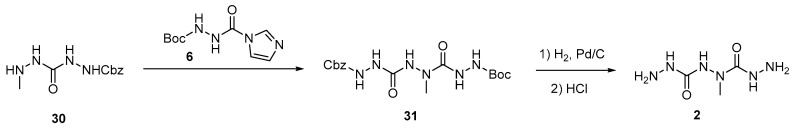
Synthesis of Gly^a^-Ala^a^-analog (**2**).

Compound **30** was used to couple the next hydrazine. Reaction of compound **30** with CDI did lead to an activated hydrazide, but this, like the other secondary hydrazides, was not reactive when coupling with another hydrazine. Therefore, compound **30** needed to be treated with an activated hydrazine (Scheme 10).

First, activated hydrazine **6** was prepared, and compound **30** was added. After stirring overnight, a complex mixture was obtained. HPLC showed that the product was present. Column chromatography afforded the desired product, **31,** in 25% yield. Since this product was still contaminated with the cyclic dimer, the material was also purified by RP-ISCO. This procedure afforded a clean compound **31**. Cbz deprotection by hydrogenation followed by Boc deprotection with HCl afforded compound **2**.

Below, we provide a detailed description of the synthetic procedures for the key intermediates utilized in the synthesis of target azatide (**2**).

#### 4.2.1. Synthesis of Tert-Butyl 2-(2-((Benzyloxy)carbonyl)hydrazine-1-carbonyl)-1-methylhydrazine-1-carboxylate (**29**)

CDI (0.6 g, 1 equiv., 3 mmol) was dissolved in 10 mL of DCM, and tert-butyl 1-methylhydrazine-1-carboxylate (**19**) (0.5 g, 1 equiv., 3 mmol) was added dropwise to 5 mL of DCM at room temperature over 10 min. The mixture was stirred for 1 h at room temperature. Benzyl hydrazinecarboxylate (**7**) (0.6 g, 1 equiv., 3 mmol) was added, and the mixture was stirred for 18 h. The mixture was concentrated, and the crude material was purified by column chromatography (silica (40 g); Heptanes/EtOAc (0–94%)). Fractions 46–53 were concentrated, affording tert-butyl 2-(2-((benzyloxy)carbonyl)hydrazine-1-carbonyl)-1-methylhydrazine-1-carboxylate (**29**) (800 mg, 2.36 mmol, 70%) as white foam.

#### 4.2.2. Synthesis of Benzyl 2-(2-Methylhydrazine-1-carbonyl)hydrazine-1-carboxylate (**30**)

Tert-butyl 2-(2-((benzyloxy)carbonyl)hydrazine-1-carbonyl)-1-methylhydrazine-1-carboxylate (**29**) (800 mg, 1 equiv., 2.36 mmol) was dissolved in 10 mL of MeOH. 5 N HCl in IPA (20 mL) was added. The mixture was stirred for 3 h at room temperature. The mixture was concentrated, affording benzyl 2-(2-methylhydrazine-1-carbonyl)hydrazine-1-carboxylate hydrochloride as a white solid. A total of 25 mL of EtOAc was added to the solid, and the mixture was neutralized with 4 mL of 1 N NaOH. The organic layer was separated and dried over Na_2_SO_4_. Concentration of the solvent afforded benzyl 2-(2-methylhydrazine-1-carbonyl)hydrazine-1-carboxylate (**30**) (490 mg, 2.06 mmol, 87.0%) as a white solid.

#### 4.2.3. Synthesis of Benzyl 2-(2-(2-(Tert-butoxycarbonyl)hydrazine-1-carbonyl)-2-methylhydrazine-1-carbonyl)hydrazine-1-carboxylate (**31**)

CDI (2.7 g, 1 equiv., 16 mmol) was dissolved in 50 mL of CH_3_CN, and tert-butyl hydrazinecarboxylate (**5**) (2.2 g, 1 equiv., 16 mmol) was added to 15 mL of CH_3_CN over 15 min to prepare compound **6**. The mixture was stirred for 1 h at room temperature. Compound benzyl 2-(2-methylhydrazine-1-carbonyl)hydrazine-1-carboxylate (**30**) (3.9 g, 1 equiv., 16 mmol) was added, and the mixture was stirred for 18 h. The mixture was concentrated. The residue was taken up in EtOAc (100 mL) and washed with 0.1 N HCl (25 mL) and brine, dried over Na_2_SO_4_ and concentrated. The crude material was purified by column chromatography (silica (120 g); DCM/MeOH (0–6%)). Fractions 37–75 were concentrated, affording 1.7 g of the product as a white foam, which was contaminated with cyclic dimer. This material was purified by RP-ISCO in 7 batches (RP (PG-45); 10 mM NH_4_HCO_3_/Acetonitrile (0–40%)). The fractions with product (for example, run 6, fractions 36–38) were combined and concentrated. The residue was dissolved in 100 mL of EtOAc, filtered, dried over Na_2_SO_4_ and concentrated, affording 200 mg of the product as a white solid. This was the desired product, but it was not entirely pure, and the recovery was low. The insoluble white solid that was filtered off was analyzed and found to be pure benzyl 2-(2-(2-(tert-butoxycarbonyl)hydrazine-1-carbonyl)-2-methylhydrazine-1-carbonyl)hydrazine-1-carboxylate (**31**) (1.0 g, 2.5 mmol, 15%).

#### 4.2.4. Synthesis of Gly^a^-Ala^a^-Analog (**2**)

Benzyl 2-(2-(2-(tert-butoxycarbonyl)hydrazine-1-carbonyl)-2-methylhydrazine-1-carbonyl)hydrazine-1-carboxylate (**31**) (1.0 g, 1 equiv., 2.5 mmol) was dissolved in 50 mL of MeOH and added to Pd/C (0.13 g, 10% wt, 0.05 equiv., 0.13 mmol).

The mixture was stirred for 1 h under a H_2_ atmosphere (balloon). The mixture was filtered over Celite, and 20 mL of 5 N HCl in IPA was added. The mixture was stirred for 2 h and then concentrated. The residue was taken up in 25 mL of water and lyophilized to remove the IPA completely. The product was obtained as a yellowish solid with a trace of IPA left. The residue was taken up in 25 mL of water and lyophilized again, affording Gly^a^-Ala^a^ dihydrochloride salt (540 mg, 2.30 mmol, 91%) as a yellowish solid.

### 4.3. Synthesis of the Gly^a^-Gly^a^-Analog (***3***)

**Scheme 11:**
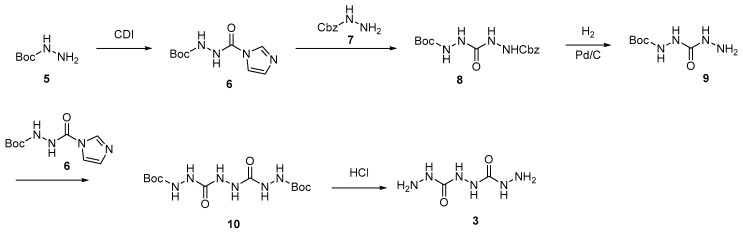
Synthesis of Gly^a^-Gly^a^-analog (**3**).

In the literature, pentafluorophenol carbamate has been used to couple two hydrazine moieties [47]. In our approach, CDI was used to couple two hydrazine moieties. Instead of the linear homopolymer (MPEG) protecting group, we tried to use Boc and Cbz as protecting groups (Scheme 11).

CDI was treated with a portion-wise addition of Boc-hydrazine **5**. The activated intermediate **6** was obtained and directly treated with Cbz-hydrazine **7**. This afforded carbazide **8**, which was purified by column chromatography. Deprotection with H_2_ and Pd/C gave compound **9**. Reaction of compound **9** with compound **6** gave compound **10**. When the coupling was performed in CH_3_CN as a solvent, the desired compound, **10,** precipitated as a pure product from the reaction mixture. A final deprotection with HCl in MeOH/IPA provided the Gly^a^-Gly^a^-analog **3**. Analysis was a bit difficult, since the compound had no UV signal on HPLC and had no identifiable protons for ^1^H-NMR. ^13^C-NMR did show the expected signal. Tiny contaminant peaks from some impurities could have emerged from the solvents used during the synthetic procedure.

Below, we provide a detailed description of the synthetic procedures for key intermediates utilized in the synthesis of target azatide (**3**).

#### 4.3.1. Synthesis of Benzyl 2-(2-(Tert-butoxycarbonyl)hydrazine-1-carbonyl)hydrazine-1-carboxylate (**8**)

CDI (2.5 g, 1 equiv., 15 mmol) was dissolved in 25 mL of DMF, and N-Boc-hydrazine (**5**) (2.0 g, 1.7 mL, 1 equiv., 15 mmol) was added portion-wise at room temperature. The mixture was stirred for 3 h at room temperature. Cbz-hydrazine **7** (2.5 g, 1 equiv., 15 mmol) was added. The mixture was stirred overnight at room temperature. The mixture was diluted with 100 mL of water and extracted with MTBE (3 × 50 mL). The organic layers were washed with water (2 × 25 mL), dried over Na_2_SO_4,_ and concentrated, affording benzyl 2-(2-(tert-butoxycarbonyl)hydrazine-1-carbonyl)hydrazine-1-carboxylate (**8**) (2.8 g, 8.6 mmol, 57%) as a white foam.

#### 4.3.2. Synthesis of Tert-Butyl 2-(Hydrazinecarbonyl)hydrazine-1-carboxylate (**9**)

Benzyl 2-(2-(tert-butoxycarbonyl)hydrazine-1-carbonyl)hydrazine-1-carboxylate (**8**) (850 mg, 1 equiv., 2.62 mmol) was dissolved in 20 mL of MeOH and added to Pd/C (139 mg, 10% wt, 0.05 equiv., 131 μmol). The mixture was stirred under a H_2_ atmosphere (balloon) for 1 h, filtered over Celite, and concentrated, affording tert-butyl 2-(hydrazinecarbonyl)hydrazine-1-carboxylate (**9**) (500 mg, 2.63 mmol, 100%) as a white foam.

#### 4.3.3. Synthesis of Gly^a^-Gly^a^-Analog (**3**)

Di-tert-butyl 2,2′-(hydrazine-1,2-dicarbonyl)bis(hydrazine-1-carboxylate) (**10**) (750 mg, 1 equiv., 2.15 mmol) was suspended in 5 mL of MeOH, and 5 mL of 6 N HCl in IPA was added. The mixture was stirred for 3 h. Since the reaction was not complete, more MeOH (40 mL) was added to dissolve the starting material. The mixture was stirred overnight. The mixture was concentrated in vacuo, affording the product as a white solid. The material was dissolved in 20 mL of water and lyophilized to remove the last traces of solvents. This afforded N’-hydrazinecarbonylmethanedihydrazide dihydrochloride (450 mg, 2.04 mmol, 94.6%) as a white solid.

### 4.4. Synthesis of the Ala^a^-Ala^a^-Analog (***4***)

**Scheme 12:**
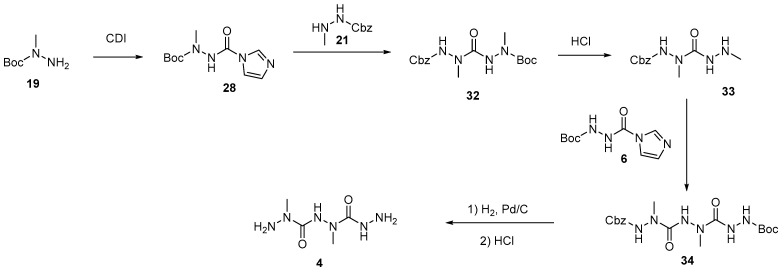
Synthesis of Ala^a^-Ala^a^-analog (**4**).

Based on the results of the previous targets, the synthetic route to compound **4** depicted in Scheme 12 was considered the most promising.

Boc-1-methylhydrazine (**19**) was treated with CDI, and activated hydrazide **28** was obtained. A trace of bis-hydrazide was also observed. Cbz-2-methylhydrazine (**21**) was added, and the desired product, **32,** was obtained after column chromatography. The Boc-group was cleaved using HCl, and free hydrazide **33** was obtained after a basic work-up. Reaction of compound **33** with activated Boc-hydrazine **6** afforded compound **34**. Column chromatography with DCM/MeOH afforded the desired product, but it was still contaminated with a cyclic dimer. Column chromatography with EtOAc/Hept afforded pure compound **34**. A final deprotection, first with H_2_ Pd/C to remove the Cbz and then with HCl, afforded the desired Ala^a^-Ala^a^-analog **4**. The product was lyophilized twice to remove IPA and MeOH completely.

Below, we provide a detailed description of the synthetic procedures for key intermediates utilized in the synthesis of target azatide (**4**).

#### 4.4.1. Synthesis of Benzyl 2-(2-(Tert-butoxycarbonyl)hydrazine-1-carbonyl)-2-methylhydrazine-1-carboxylate (**22**) as a Potential Common Intermediate for Azatides (**2**) and (**4**)

CDI (2.5 g, 1 equiv., 15 mmol) was dissolved in 50 mL of CH_3_CN, and N-Boc-hydrazine (**5**) (2.0 g, 1.7 mL, 1 equiv., 15 mmol) was added dropwise to 20 mL of CH_3_CN at room temperature. The mixture was stirred for 1.5 h at room temperature. Benzyl 2-methylhydrazine-1-carboxylate (**21**) (2.7 g, 1 equiv., 15 mmol) was added. The mixture was stirred for 20 h at room temperature and concentrated. The crude material was purified by means of column chromatography (silica (80 g); Heptanes/EtOAc (0–70%)). Fractions 68–94 were concentrated, affording benzyl 2-(2-(tert-butoxycarbonyl)hydrazine-1-carbonyl)-2-methylhydrazine-1-carboxylate (**22**) (2.9 g, 8.6 mmol, 57%) as a white foam.

#### 4.4.2. Synthesis of Tert-Butyl 2-(1-Methylhydrazine-1-carbonyl)hydrazine-1-carboxylate (**23**) as a Potential Common Intermediate for Azatides (**2**) and (**4**)

Benzyl 2-(2-(tert-butoxycarbonyl)hydrazine-1-carbonyl)-2-methylhydrazine-1-carboxylate (**22**) (2.9 g, 1 equiv., 8.6 mmol) was dissolved in 50 mL of MeOH and added to Pd/C (0.46 g, 10% wt, 0.05 equiv., 0.43 mmol). The mixture was stirred under a H_2_ atmosphere (balloon) for 1 h. The mixture was filtered over Celite, concentrated, and stripped with DCM to remove MeOH completely. Tert-butyl 2-(1-methylhydrazine-1-carbonyl)hydrazine-1-carboxylate (**23**) (1.8 g, 8.8 mmol, 100%) was isolated as a white solid.

#### 4.4.3. Synthesis of Benzyl 2-(2-(Tert-butoxycarbonyl)-2-methylhydrazine-1-carbonyl)-2-methylhydrazine-1-carboxylate (**32**)

CDI (2.49 g, 1.0 equiv., 15.4 mmol) was dissolved in 10 mL of DCM, and tert-butyl 1-methylhydrazine-1-carboxylate (**19**) (2.25 g, 1.0 equiv., 15.4 mmol) was added dropwise to 20 mL of DCM at room temperature over 10 min. The mixture was stirred for 1 h at room temperature. Benzyl 2-methylhydrazine-1-carboxylate (**21**) (2.77 g, 1.0 equiv., 15.4 mmol) was added, and the mixture was stirred overnight. The mixture was diluted with 50 mL of DCM and washed with 0.1 N HCl (2 × 10 mL and brine, dried over Na_2_SO_4,_ and concentrated. The crude material was purified by means of column chromatography (silica (120 g); Heptanes/EtOAc (0–60%)). Fractions 76–123 were concentrated, affording benzyl 2-(2-(tert-butoxycarbonyl)-2-methylhydrazine-1-carbonyl)-2-methylhydrazine-1-carboxylate (**32**) (2.8 g, 7.9 mmol, 52%) as a white wax.

#### 4.4.4. Synthesis of Benzyl 2-Methyl-2-(2-methylhydrazine-1-carbonyl)hydrazine-1-carboxylate (**33**)

Benzyl 2-(2-(tert-butoxycarbonyl)-2-methylhydrazine-1-carbonyl)-2-methylhydrazine-1-carboxylate (**32**) (2.8 g, 1 equiv., 7.9 mmol) was dissolved in 20 mL of MeOH, and 30 mL of 5 N HCl in IPA was added. The mixture was stirred for 1 h at room temperature. The mixture was concentrated, then 50 mL of EtOAc and 5 mL of brine were added to the solid. The mixture was neutralized with 8 mL of 1 N NaOH. The organic layer was separated and dried over Na_2_SO_4_. Concentration of the solvent afforded benzyl 2-methyl-2-(2-methylhydrazine-1-carbonyl)hydrazine-1-carboxylate (**33**) (1.8 g, 7.1 mmol, 90%) as a colorless oil that held on to the solvent.

#### 4.4.5. Synthesis of Benzyl 2-(2-(2-(Tert-butoxycarbonyl)hydrazine-1-carbonyl)-2-methylhydrazine-1-carbonyl)-2-methylhydrazine-1-carboxylate (**34**)

CDI (1.2 g, 1.0 equiv., 7.1 mmol) was dissolved in 50 mL of DCM, and tert-butyl hydrazinecarboxylate (**5**) (0.94 g, 1.0 equiv., 7.1 mmol) was added to 15 mL of DCM over 15 min to prepare compound **6**. The mixture was stirred for 1 h at room temperature. Benzyl 2-methyl-2-(2-methylhydrazine-1-carbonyl)hydrazine-1-carboxylate (**33**) (1.8 g, 1.0 equiv., 7.1 mmol) was added, and the mixture was stirred for 18 h. The mixture was washed with 0.1 N HCl (2 × 25 mL) and brine, dried over Na_2_SO_4,_ and concentrated. The crude material was purified by means of column chromatography (silica (80 g); DCM/MeOH (0–6%)). Fractions 53–90 were concentrated, affording 1.7 g of the product as a white solid. Since the resulting product was not pure enough, it was additionally purified by column chromatography (silica (40 g); Heptanes/EtOAc (0–100%)). Fractions 60–78 were concentrated, affording benzyl 2-(2-(2-(tert-butoxycarbonyl)hydrazine-1-carbonyl)-2-methylhydrazine-1-carbonyl)-2-methylhydrazine-1-carboxylate (**34**) (1.4 g, 3.4 mmol, 48%) as a white solid.

#### 4.4.6. Synthesis of Ala^a^-Ala^a^-Analog (**4**)

Benzyl 2-(2-(2-(tert-butoxycarbonyl)hydrazine-1-carbonyl)-2-methylhydrazine-1-carbonyl)-2-methylhydrazine-1-carboxylate (**34**) (1.4 g, 1 equiv., 3.4 mmol) was dissolved in 50 mL of MeOH and added to Pd/C (0.18 g, 10% wt, 0.05 equiv., 0.17 mmol). The mixture was stirred for 1 h under a H_2_ atmosphere (balloon). The mixture was filtered over Celite, and 20 mL of 5 N HCl in IPA was added. The mixture was stirred for 3 h and concentrated.

The residue was taken up in 25 mL of water and lyophilized, affording 800 mg of the product as a white solid. A trace of IPA was left. The product was taken up in 25 mL of water and lyophilized again, affording N’-hydrazinecarbonyl-N,N’’-dimethylmethanedihydrazide dihydrochloride (800 mg, 3.21 mmol, 94%) as a white solid.

We confirmed the structural integrity of azatides **1**–**4** by ^13^C and ^1^H NMR in D_2_O at room temperature (Figure 6 and Figure 7).

## 5. Conclusions

We synthesized four azatide dipeptide analogs, Ala^a^-Gly^a^ (**1**), Gly^a^-Ala^a^ (**2**), Gly^a^-Gly^a^ (**3**), and Ala^a^-Ala^a^ (**4**), as potential candidate backbone motifs for peptide-like polymers that could persist in planetary environments dominated by concentrated sulfuric acid, such as the clouds of Venus [65] or, potentially, exoplanets [68]. We systematically evaluated their stability and reactivity in 98% *w*/*w* sulfuric acid using ^1^H and ^13^C NMR spectroscopy, supported by ELSD-LCMS measurements.

We show that all four azatides readily dissolve in 98% *w*/*w* D_2_SO_4_ and are generally stable at room temperature after two weeks of incubation in 98% *w*/*w* sulfuric acid. Notably, Gly^a^-Ala^a^ (**2**) displays no detectable degradation over a two-week period. Qualitative NMR and ELSD-LCMS experiments show that the remaining analogs, Ala^a^-Gly^a^ (**1**), Gly^a^-Gly^a^ (**3**), and Ala^a^-Ala^a^ (**4**), undergo only minor to moderate decomposition under the same conditions.

We emphasize, however, that this rt stability does not extend to elevated temperatures, which are representative of the lower Venusian cloud decks. At 50 °C, all four tested compounds experience significant decomposition. At 80 °C, complete degradation occurs within one to two weeks for all analogs.

We conclude that while azatides offer promising chemical stability in 98% *w*/*w* sulfuric acid at temperate conditions, their thermal instability at higher temperatures severely limits their viability as alternative backbone motifs for biological polymers capable of persisting under the full temperature gradient of Venus’ clouds.

Future work, both experimental as well as computational and theoretical modeling, could elucidate the exact mechanism of solvolysis of azatides in concentrated sulfuric acid, investigate potential stabilizing modifications to the azatide backbone, or model the conformational and dynamical range of the rigid, nitrogen-rich azatide structure.

## Figures and Tables

**Table 1:** Summary of combined NMR and ELSD-LCMS results of the qualitative stability assessment of the azatide dipeptide analogs (**1**–**4**) after a two-week incubation in 98% *w*/*w* sulfuric acid at rt, 50 °C and 80 °C. Color shading corresponds to the degree of compound degradation in concentrated sulfuric acid. No degradation (dark green), minor degradation (light green), moderate degradation (light pink), large degradation (dark pink), complete degradation (red).

	Name	Structure	RT	50 °C	80 °C
**1**	Ala^a^-Gly^a^	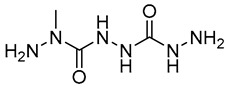	Minor degradation	Moderate degradation	Full degradation
**2**	Gly^a^-Ala^a^	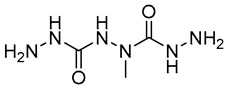	No degradation	Minor degradation	Full degradation
**3**	Gly^a^-Gly^a^	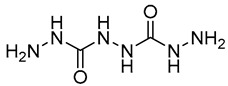	Minor degradation	Moderate degradation	Full degradation
**4**	Ala^a^-Ala^a^	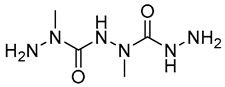	Minor degradation	Large degradation	Full degradation

**Table 2:** Qualitative stability assessment of Ala^a^-Gly^a^ azatide (**1**) in 98% *w*/*w* D_2_SO_4_ with ELSD ^1^. Color shading corresponds to the degree of compound degradation in concentrated sulfuric acid. No degradation (dark green), minor degradation (light green), moderate degradation (light pink), complete degradation (red).

Incub. Temp./Time	1 h	24 h	7 d	14 d
98% *w*/*w* D_2_SO_4_ at RT	No degradation	No degradation	No degradation	Minor degradation
98% *w*/*w* D_2_SO_4_ at 50 °C	No degradation	No degradation	Minor degradation	Moderate degradation
98% *w*/*w* D_2_SO_4_ at 80 °C	No degradation	Full degradation	Full degradation	Full degradation

^1^ Reaction conditions: 11 mg of azatide **1** dihydrochloride was dissolved in 1.1 mL 98% *w*/*w* D_2_SO_4_ (2 wt% D_2_O) and stirred for the given time at the given temperature. The material was very hygroscopic, which might influence the stability estimate. A total of 0.1 mL of the mixture was taken for each measurement and added to 0.9 mL of a 3M NH_4_OAc (in water) buffer, resulting in a pH of 2–3. The samples were analyzed by LCMS-ELSD.

**Table 3:** Qualitative stability assessment of Gly^a^-Ala^a^ azatide (**2**) in 98% *w*/*w* D_2_SO_4_ with ELSD ^1^. Color shading corresponds to the degree of compound degradation in concentrated sulfuric acid. No degradation (dark green), minor degradation (light green), complete degradation (red).

Incub. Temp./Time	1 h	24 h	7 d	14 d
98% *w*/*w* D_2_SO_4_ at RT	No degradation	No degradation	No degradation	No degradation
98% *w*/*w* D_2_SO_4_ at 50 °C	No degradation	No degradation	Minor degradation	Minor degradation
98% *w*/*w* D_2_SO_4_ at 80 °C	No degradation	Minor degradation	Full degradation	Full degradation

^1^ Reaction conditions: 11 mg of azatide **2** dihydrochloride was dissolved in 1.1 mL 98% *w*/*w* D_2_SO_4_ (2 wt% D_2_O) and stirred for the given time at the given temperature. The material was very hygroscopic, which might influence the stability estimate. A total of 0.1 mL of the mixture was taken for each measurement and added to 0.9 mL of a 3M NH_4_OAc (in water) buffer, resulting in a pH of 2–3. The samples were analyzed by LCMS-ELSD.

**Table 4:** Qualitative stability assessment of Gly^a^-Gly^a^ azatide (**3**) in 98% *w*/*w* D_2_SO_4_ with ELSD ^1^. Color shading corresponds to the degree of compound degradation in concentrated sulfuric acid. No degradation (dark green), minor degradation (light green), moderate degradation (light pink), complete degradation (red).

Incub. Temp./Time	1 h	24 h	7 d	14 d
98% *w*/*w* D_2_SO_4_ at RT	No degradation	No degradation	Minor degradation	Minor degradation
98% *w*/*w* D_2_SO_4_ at 50 °C	No degradation	Minor degradation	Minor degradation	Moderate degradation
98% *w*/*w* D_2_SO_4_ at 80 °C	No degradation	Minor degradation	Full degradation	Full degradation

^1^ Reaction conditions: 11 mg of azatide **3** dihydrochloride was dissolved in 1.1 mL 98% *w*/*w* D_2_SO_4_ (2 wt% D_2_O) and stirred for the given time at the given temperature. The material was very hygroscopic, which might influence the stability estimate. A total of 0.1 mL of the mixture was taken for each measurement and added to 0.9 mL of a 3M NH_4_OAc (in water) buffer, resulting in a pH of 2–3. The samples were analyzed by LCMS-ELSD.

**Table 5:** Qualitative stability assessment of Ala^a^-Ala^a^ azatide (**4**) in 98% *w*/*w* D_2_SO_4_ with ELSD ^1^. Color shading corresponds to the degree of compound degradation in concentrated sulfuric acid. No degradation (dark green), minor degradation (light green), moderate degradation (light pink), large degradation (dark pink), complete degradation (red).

Incub. Temp./Time	1 h	24 h	7 d	14 d
98% *w*/*w* D_2_SO_4_ at RT	No degradation	No degradation	No degradation	No degradation
98% *w*/*w* D_2_SO_4_ at 50 °C	Minor degradation	Moderate degradation	Large degradation	Large degradation
98% *w*/*w* D_2_SO_4_ at 80 °C	Large degradation	Large degradation	Full degradation	Full degradation

^1^ Reaction conditions: 11 mg of azatide **4** dihydrochloride was dissolved in 1.1 mL 98% *w*/*w* D_2_SO_4_ (2 wt% D_2_O) and stirred for the given time at the given temperature. The material was very hygroscopic, which might influence the stability estimate. A total of 0.1 mL of the mixture was taken for each measurement and added to 0.9 mL of a 3M NH_4_OAc (in water) buffer, resulting in a pH of 2–3. The samples were analyzed by LCMS-ELSD.

## Data Availability

All data needed to evaluate the conclusions in the paper are present in the paper and available for download from Zenodo at https://zenodo.org/records/18442323.

## Supplementary Materials

The following supporting information can be downloaded at: https://zenodo.org/records/18442323.

## Abbreviations

The following abbreviations are used in this manuscript:

NMR	Nuclear magnetic resonance spectroscopy
LCMS	Liquid Chromatography–Mass Spectrometry
ELSD	Evaporative Light Scattering Detector
rt	Room temperature
